# 

**Published:** 1869-01-01

**Authors:** 


### *CONTENTS:*

ORIGINAL PAPERS:

Pathological Chemistry — Researches into the Elimination of the Bromides, &c. By Dr. Rabuteau .... 1
A Case of Fibrous Cancer of the Stomach. By R. G. Bogue, M.D., Surgeon to Cook County Hospital, Chicago .... 11
A Child without Calvaria or Brain. Reported by L. Redmond, M.D., Preston, Minn .... 15
How does Gelseminum Destroy Life? By Z. C. McElroy, M.D., Zanesville, Ohio .... 16
Uterine Displacements. By H. Webster Jones, A.M., M.D. .... 20

CORRESPONDENCE .... 24

BOOK NOTICES:

De la Cantharide Officinale These de Pharmacie .... 25
Annual Report of the Surgeon General, United States Army, 1868 .... 28
A Treatise on Physiology and Hygiene .... 28
A Hand-Book of Vaccination. By Edward C. Leaton, M.D .... 29
The Opium Habit; with suggestions as to the Remedy .... 29
Lectures on the Study of Fever. By Alfred Hudson, M.D., M.R.I.A. .... 29
Books and Pamphlets Received .... 30

EDITORIAL:

Carroll County Medical Society .... 31

